# Supplementary material for: Manganese-driven CoQ deficiency
Source: Nat Commun. 2022 Oct 13;13:6061. doi: 10.1038/s41467-022-33641-x (PMC9563070; doi:10.1038/s41467-022-33641-x)
Supplement: Supplementary file 1 — Supplementary Information [file 41467_2022_33641_MOESM1_ESM.pdf]

Supplementary Information for

Manganese-driven CoQ deficiency

Jutta Diessl[1], Jens Berndtsson[2], Filomena Broeskamp[1], Lukas Habernig[1], Verena Kohler[1], Carmela Vazquez-Calvo[1,2], Arpita Nandy[3,4,5], Carlotta Peselj[1], Sofia Drobysheva[1], Ludovic Pelosi[6], F.-Nora Vögtle[3,7,8,9], Fabien Pierrel[6], Martin Ott[2,10], Sabrina Büttner[1,*]

[1] Department of Molecular Biosciences, The Wenner-Gren Institute, Stockholm University; 10691 Stockholm, Sweden.

[2] Department of Biochemistry and Biophysics, Stockholm University; 10691 Stockholm, Sweden.

[3] Institute of Biochemistry and Molecular Biology, ZBMZ, University of Freiburg; 79104 Freiburg, Germany.

[4] Faculty of Biology, University of Freiburg; 79104 Freiburg, Germany.

[5] Spemann Graduate School of Biology and Medicine, University of Freiburg; 79104 Freiburg, Germany.

[6] Univ. Grenoble Alpes, CNRS, UMR 5525, VetAgro Sup, Grenoble INP, TIMC; 38000 Grenoble, France.

[7] CIBSS - Centre for Integrative Biological Signalling Studies, University of Freiburg; 79104 Freiburg, Germany.

[8] Center for Molecular Biology of Heidelberg University (ZMBH), DKFZ-ZMBH Alliance; 69120 Heidelberg, Germany.

[9] Network Aging Research, Heidelberg University; 69120 Heidelberg, Germany

[10] Department of Medical Biochemistry and Cell Biology, Institute of Biomedicine, University of Gothenburg; 40530 Gothenburg, Sweden.

** Correspondence. E-mail: sabrina.buettner@su.se*

Supplementary Information includes:

Supplementary Figure 1. Cellular Mn overload results in accumulation of Mn within mitochondria, reduced mitochondrial mass and extra-mitochondrial accumulation of reactive oxygen species.

Supplementary Figure 2. Overexpression of Coq4 does not prevent Mn-driven respiratory defects.

Supplementary Figure 3. Proteins associated with CoQ metabolism are not generally deregulated upon Mn overload.

Supplementary Figure 4. Mn overload causes destabilization of Coq7.

Supplementary Figure 5. Gating strategies for flow cytometry.

Supplementary Table 1. Total cellular Mn levels in yeast mutants lacking proteins involved in Mn homeostasis.

Supplementary Table 2. Total cellular metal concentrations in cells lacking Pmr1.

Supplementary Table 3. Yeast strains used in this study.

Supplementary Table 4. Plasmids used in this study.

Supplementary Table 5. Oligonucleotides used for gene deletion, chromosomal tagging and introduction of point mutations.

Supplementary Table 6. Oligonucleotides used to construct plasmids.

Supplementary Table 7. Instrument specifications S2 Picofox spectrometer

Supplementary Table 8. Oligonucleotides used in this study for qRT-PCR.

Supplementary Table 9. Details of statistical analyses performed.

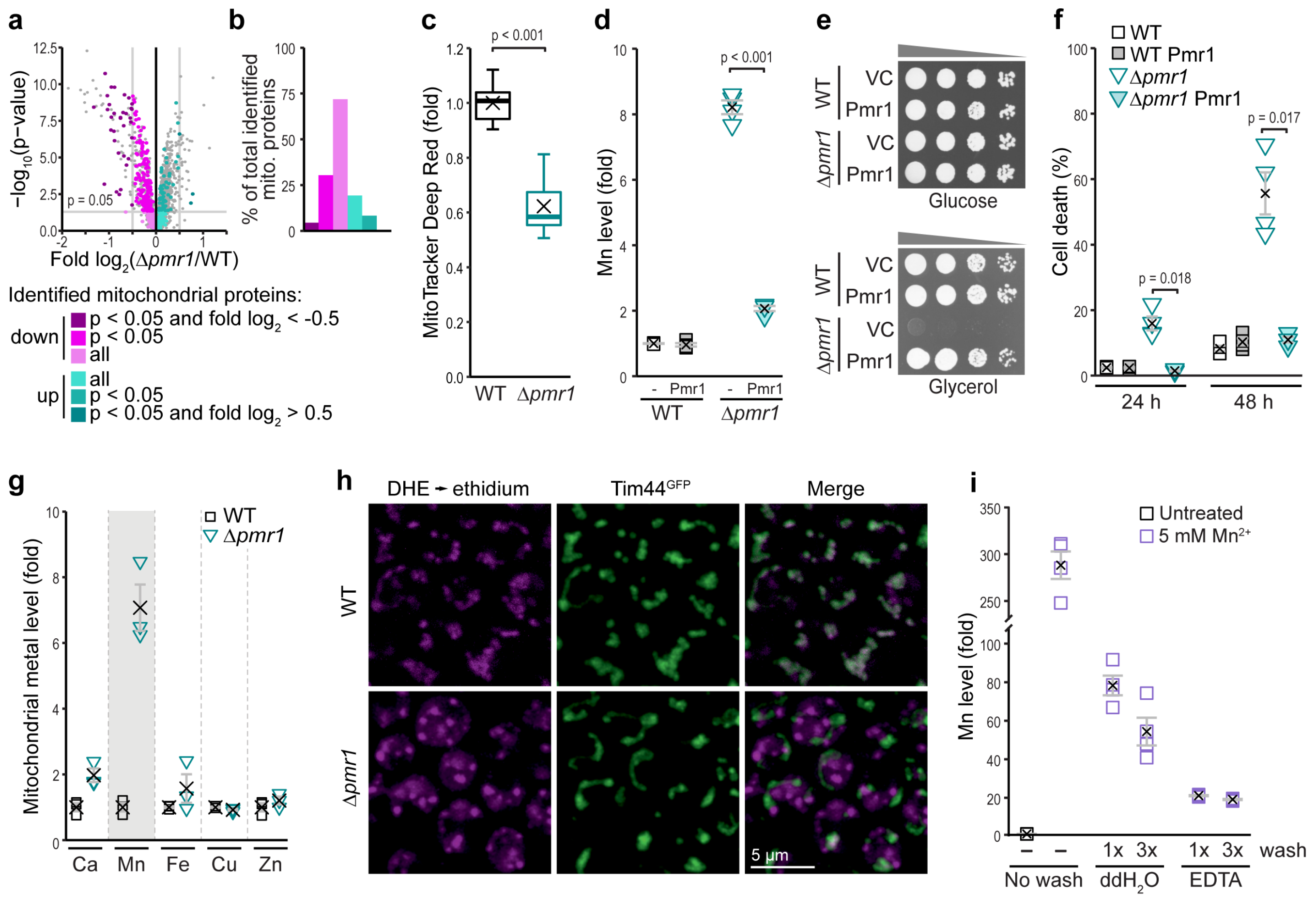

Supplementary Figure 1: Cellular Mn overload results in accumulation of Mn within mitochondria, reduced mitochondrial mass and extra-mitochondrial accumulation of reactive oxygen species.

(a, b) Volcano plot (a) and clustering of all mitochondrial proteins identified (b) in whole cell proteomics of wild type (WT) and Δ*pmr1* cells, n = 4 (WT) or 3 (Δ*pmr1*). All mitochondrial proteins identified are highlighted in color in (a) and have been further clustered into decreased abundance (down) and increased abundance (up) as well as significance and fold change values (b) as indicated. **(c)** Flow cytometric quantification of MitoTracker Deep Red staining, a mitochondrial transmembrane potential-independent fluorescent dye indicative of mitochondrial mass, of WT and Δ*pmr1* cells grown for 24 h on glucose. Box plot shows mean (x), median (line), first/third quartile (lower/upper bound), minimum/maximum within 1.5-fold IQR (lower/upper whisker) and outliers outside 1.5-fold IQR (o); n = 8. (**d**) Total cellular Mn content of WT and Δ*pmr1* cells ectopically expressing Pmr1 under control of its native promoter, determined via Total Reflection X-Ray Fluorescence Spectrometry (TXRF). Cells were collected after 24 h of growth on glucose media. Means ± SEM, n = 4. (**e**) Spotting of serial dilutions of cells described in (d) on glucose and glycerol media. (**f**) Survival determined by flow cytometric quantification of propidium iodide staining of cells described in (d) at 24 h and 48 h of growth on glucose media. Means ± SEM, n = 4. **(g)** Metal content of mitochondria isolated from WT and Δ*pmr1* cells determined via TXRF. Means ± SEM, n = 3. **(h)** Representative confocal micrographs (of n = 3) visualizing the production of reactive oxygen species in WT and Δ*pmr1* cells endogenously expressing a Tim44-GFP chimera to visualize mitochondria. Cells were grown for 24 h and stained with dihydroethidium (DHE), which is converted to fluorescent ethidium by reactive oxygen species. (**i**) Total cellular Mn content of WT cells treated with 5 mM Mn^{2+} for 24 h. To control for unspecific binding of Mn to the cell surface, cells have been washed either with ddH_20 (1x or 3x wash) or with 10 mM EDTA (1x or 3x wash). Values were normalized to untreated cells. Means ± SEM, n = 4. Details for statistical analysis (Supplementary Table 9) and source data are provided.

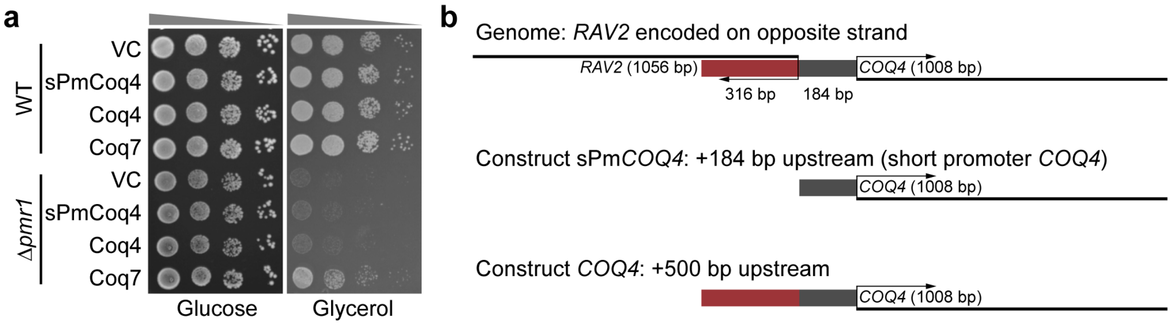

Supplementary Figure 2. Overexpression of Coq4 does not prevent Mn-driven respiratory defects.
(a) Spotting of serial dilutions of WT and Δ*pmr1* cells harboring the vector control (VC) or overexpressing Coq4 and Coq7 on plates containing glucose or glycerol as indicated. **(b)** Schematic of the constructs used in (a). sPmCoq4 is a construct with a shorter region (184 bp) before the initial ATG of the *COQ4* coding sequence to control for confounding effects of the *RAV2* gene fragment on the opposite strand. Constructs Coq4 and Coq7 refer to constructs that encode the respective coding sequences + 500 bp upstream of their initial ATGs.

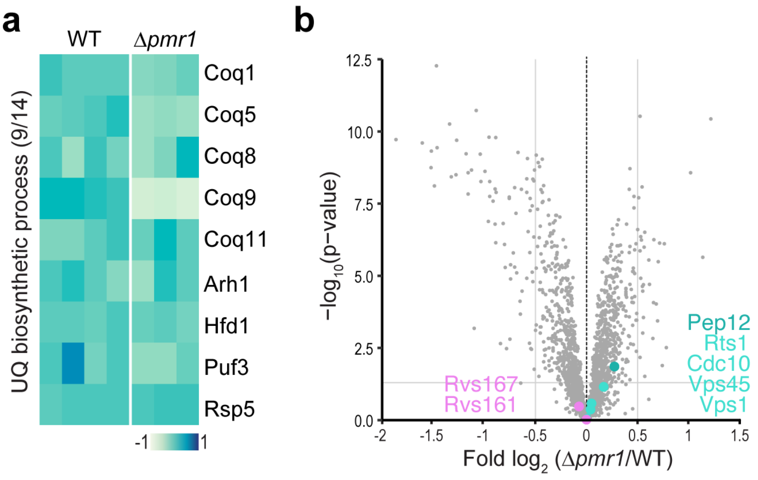

Supplementary Figure 3. Proteins associated with CoQ metabolism are not generally deregulated upon Mn overload.
(a) Heatmap of all identified proteins associated with the GO term 'ubiquinone biosynthetic process' from whole cell proteomics of WT and Δ*pmr1* cells (corresponding to Fig. 1e, f). n = 4 (WT) or 3 (Δ*pmr1*). **(b)** Volcano plot displaying results of differential protein abundance analysis of whole cell proteomics. Proteins connected to CoQ uptake and intracellular transport are labeled. Complete DeqMS analysis of proteomics data is available in Supplementary Data 1, and raw data are provided in the Source Data file.

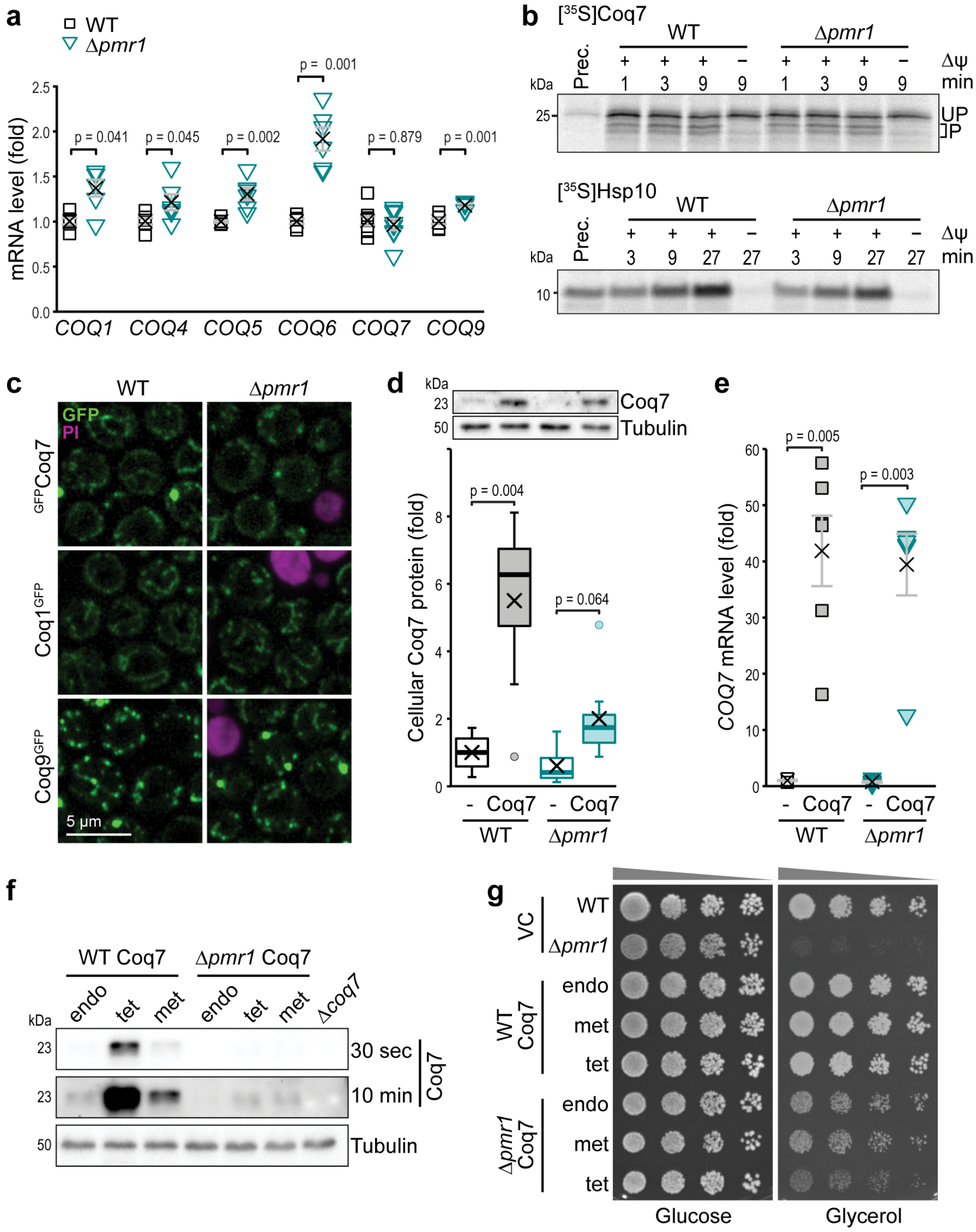

Supplementary Figure 4: Mn overload causes destabilization of Coq7.

(a) qRT-PCR-based quantification of mRNA levels of *COQ1*, *COQ4*, *COQ5*, *COQ6*, *COQ7* and *COQ9* in WT and Δ*pmr1* cells grown for 24 h on glucose media. *UBC6* was used as the housekeeping gene. Means ± SEM, n = 6-8. **(b)** Autoradiograms of *in organello* import assays into mitochondria isolated from WT and Δ*pmr1* cells grown for 24 h on glucose media (representative images of n = 3 are shown). Radiolabeled Coq7 and Hsp10 were imported for indicated times, and, where indicated, mitochondrial transmembrane potential (Δψ) was dissipated using a mix of 1 µM valinomycin, 20 µM oligomycin and 8 µM antimycin A. P = processed, UP = unprocessed. **(c)** Representative confocal micrographs (of n = 3) of WT and Δ*pmr1* cells harboring endogenously GFP-tagged Coq7, Coq1 or Coq9 grown for 24 h on glucose. Cells were counterstained with propidium iodide (PI) to exclude dead cells. **(d)** Immunoblot analysis and corresponding densitometric quantification of Coq7 protein levels in total cell lysates from WT and Δ*pmr1* cells overexpressing Coq7 or harboring the vector control. Blots were decorated with antibodies against Coq7 and tubulin as loading control. Box plot shows mean (x), median (line), first/third quartile (lower/upper bound), minimum/maximum within 1.5-fold IQR (lower/upper whisker) and outliers outside 1.5-fold IQR (o), n = 8.

(e) qRT-PCR-based quantification of mRNA levels of *COQ7* normalized to *UBC6* as housekeeping gene of cells described in (d). Means ± SEM, n = 6. **(f)** Representative immunoblot analysis (of n = 3) of total cell lysates from WT and Δ*pmr1* cells harboring plasmids overexpressing Coq7 under endogenous (endo), methionine-repressible (met) or tetracycline-repressible (tet) promoters grown for 24 h on glucose media. Cells lacking Coq7 served as control. Blots were decorated with antibodies against Coq7 and tubulin as loading control. **(g)** Spotting of serial dilutions of cells described in (f) on plates containing glucose or glycerol as indicated. Details for statistical analysis (Supplementary Table 9) and source data are provided.

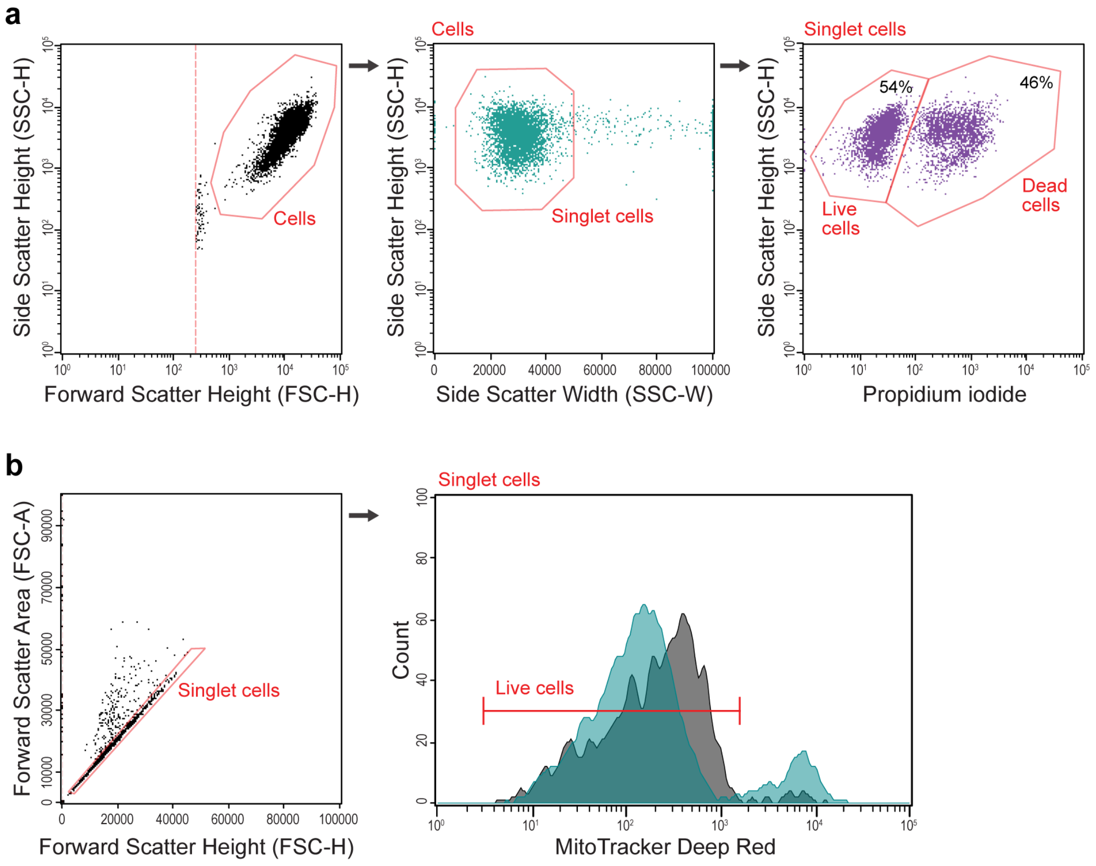

Supplementary Figure 5: Gating strategies for flow cytometry.

(a) Gating strategy for flow cytometric quantification of live and dead cells using propidium iodide (PI). Yeast cells were distinguished from debris by gating cells in Side Scatter height (SSC-H) vs Forward Scatter height (FSC-H). Doublets were discriminated from singlets by gating cells in Side Scatter height (SSC-H) vs width (SSC-W). For cell death analysis, this population was gated into PI-positive (dead) and PI-negative (live) populations. Percentage of PI-negative cells is reported as survival (Fig. 1d and 3b) and percentage of PI-positive cells is reported as cell death (Supplementary Fig. 1f). **(b)** For flow cytometric quantification of mitochondrial mass using MitoTracker Deep Red staining, singlet cells were identified by gating cells in Forward Scatter Area (FSC-A) vs FSC-H. Subsequently, the median red fluorescence intensity of the singlet cell population was divided by the FSC-H value to normalize to cell size. Gating strategy corresponds to flow cytometry data shown in Supplementary Fig. 1c.

Supplementary Table 1. Total cellular Mn levels in yeast mutants lacking proteins involved in Mn homeostasis. Concentration determined via TXRF was normalized to OD. Mean of n = 6 (WT) or n = 3 (mutants) and standard error of mean (SEM) are shown.

Genotype	Mn (ng/OD)	SEM
WT	1.60	0.05
Δ*smf2*	0.22	0.22
Δ*smf1*	0.68	0.09
Δ*mtm1*	1.09	0.11
Δ*ypk9*	1.22	0.13
Δ*yke4*	1.27	0.10
Δ*atx2*	1.37	0.06
Δ*ccc1*	1.37	0.05
Δ*pho84*	1.46	0.15
Δ*msc2*	1.49	0.09
Δ*zrc1*	1.53	0.01
Δ*ccc1*Δ*ypk9*	1.61	0.33
Δ*cot1*	1.62	0.13
Δ*smf3*Δ*ccc1*	2.19	0.13
Δ*spf1*	2.34	0.16
Δ*smf3*	2.35	0.24
Δ*smf3*Δ*ypk9*	2.45	0.01
Δ*ccc1*Δ*smf3*Δ*ypk9*	2.64	0.22
Δ*gdt1*	3.48	0.33
Δ*pmr1*	12.03	1.02

Supplementary Table 2. Total cellular metal concentrations in cells lacking Pmr1. Concentrations determined via TXRF were normalized to OD. Mean of n = 3 and standard error of mean (SEM) are shown.

Element	Genotype	Metal (ng/OD)	SEM
Ca	WT	61.15	5.90
Ca	Δ*pmr1*	132.09	1.26
Mn	WT	1.31	0.06
Mn	Δ*pmr1*	13.96	0.43
Fe	WT	5.00	0.48
Fe	Δ*pmr1*	7.91	0.34
Cu	WT	1.29	0.13
Cu	Δ*pmr1*	1.70	0.07
Zn	WT	11.34	1.07
Zn	Δ*pmr1*	17.45	0.60

Supplementary Table 3. Yeast strains used in this study.

Strain	Genotype	Source
WT (wild type)	BY4741 MATa, *his3*Δ1, *leu2*Δ0, *met15*Δ0, *ura3*Δ0	Euroscarf
Δ*smf2*	BY4741 Δ*smf2*::kanMX	Euroscarf
Δ*smf1*	BY4741 Δ*smf1*::kanMX	Euroscarf
Δ*mtm1*	BY4741 Δ*mtm1*::kanMX	Euroscarf
Δ*ypk9*	BY4741 Δ*ypk9*::natNT2	This study
Δ*yke4*	BY4741 Δ*yke4*::kanMX	Euroscarf
Δ*ccc1*	BY4741 Δ*ccc1*::natNT2	This study
Δ*ccc1*Δ*ypk9*	BY4741 Δ*ccc1*::natNT2 Δ*ypk9*::hphNT1	This study
Δ*atx2*	BY4741 Δ*atx2*::kanMX	Euroscarf
Δ*pho84*	BY4741 Δ*pho84*::kanMX	Euroscarf
Δ*msc2*	BY4741 Δ*msc2*::kanMX	Euroscarf
Δ*zrc1*	BY4741 Δ*zrc1*::kanMX	Euroscarf
Δ*cot1*	BY4741 Δ*cot1*::kanMX	Euroscarf
Δ*smf3*	BY4741 Δ*smf3*::natNT2	This study
Δ*smf3*Δ*ccc1*	BY4741 Δ*smf3*::natNT2 Δ*ccc1*::hphNT1	This study
Δ*smf3*Δ*ccc1*Δ*ypk9*	BY4741 Δ*smf3*::natNT2 Δ*ccc1*::hphNT1 Δ*ypk9*:kanMX	This study
Δ*smf3*Δ*ypk9*	BY4741 Δ*smf3*::natNT2 Δ*ypk9*::hphNT1	This study
Δ*spf1*	BY4741 Δ*spf1*::natNT2	This study
Δ*gdt1*	BY4741 Δ*gdt1*::natNT2	This study
Δ*pmr1*	BY4741 Δ*pmr1*::kanMX (*HUR1* intact)	(Diessl et al., 2020)
Δ*coq7*	BY4741 Δ*coq7*::natNT2	This study
Pmr1^{D53A}	BY4741 *pmr1*-D53A	This study
Pmr1^{Q783A}	BY4741 *pmr1*-Q783A	This study
^{GFP}Coq7	BY4741 *coq7*-nt108::yEGFP	This study
^{GFP}Coq7 Δ*pmr1*	^{GFP}Coq7 Δ*pmr1*::kanMX (*HUR1* intact)	This study
Coq1^{GFP}	BY4741 *COQ1*-yEGFP::hphNT1	This study
Coq1^{GFP} Δ*pmr1*	Coq1^{GFP} Δ*pmr1*::kanMX (*HUR1* intact)	This study
Coq9^{GFP}	BY4741 *COQ9*-yEGFP::hphNT1	This study
Coq9^{GFP} Δ*pmr1*	Coq9^{GFP} Δ*pmr1*::kanMX (*HUR1* intact)	This study
Tim44^{GFP}	BY4741 *TIM44*-GFP(S65T)-*HIS3*	GFP collection UCSF
Tim44^{GFP} Δ*pmr1*	Tim44^{GFP} Δ*pmr1*::kanMX (*HUR1* intact)	This study
TetOFF-Pim1	BY4741 *HIS3MX6*-tTA-TetO-*pCYC1*-*PIM1*	This study
TetOFF-Pim1 Δ*pmr1*	TetOFF-Pim1 Δ*pmr1*::kanMX (*HUR1* intact)	This study

Supplementary Table 4. Plasmids used in this study.

Plasmid	Description	Source
pHR81-URA-VC	Empty vector for genomic library	This study
pHR81-URA-sPmCOQ4	*COQ4* coding region + 184 bp upstream of start codon	This study
pHR81-URA-COQ4	*COQ4* coding region + 500 bp upstream of start codon	This study
pHR81-URA-COQ7	*COQ7* coding region + 500 bp upstream of start codon	This study
pHR81-URA-FLAG-COQ7	FLAG-*COQ7* coding region + 500 bp upstream of start codon	This study
pHR81-URA-GFP-COQ7	GFP-*COQ7* coding region + 500 bp upstream of start codon	This study
pHR81-URA-COQ8	*COQ8* coding region + 500 bp upstream of start codon	This study
pHR81-URA-COQ9	*COQ9* coding region + 500 bp upstream of start codon	This study
pHR81-URA-IAH1	*IAH1* coding region + 500 bp upstream of start codon	This study
pCM190-URA-VC	Empty vector with tetO-CYC1 promoter	(Garí et al., 1997)
pCM190-URA-COQ7	*COQ7* coding region under tetO-CYC1 promoter	This study
pUG35-URA-VC	Empty vector with MET25 promoter	U. Güldener and J. H. Hegemann, Heinrich-Heine-Universität, Germany
pUG35-URA-COQ7	*COQ7* coding region (including stop codon) under MET25 promoter	This study
pHR81-HIS-VC	Empty vector	This study
pHR81-HIS-COQ9	COQ9 coding region + 500 bp upstream of start codon	This study
pRS313-HIS-PMR1	*PMR1* coding region + 700 bp upstream of start codon	This study
pYM-HIS-N-tetOFF	Vector for amplification of tetracycline-regulatable promoter system (tetO7-CYC1 promoter) with Tet transactivator domain and His3MX6 selection marker	This study

Supplementary Table 5. Oligonucleotides used for gene deletion, chromosomal tagging and introduction of point mutations.

Purpose	Oligonucleotides	Template
Deletion of *PMR1*	5'-CAG CAC AGA CGT AAG CTT AAG TGT AAG TAA AAG ATA AGA TAA TCA GCT GAA GCT TCG TAC GC-3'<br>5'-TAA CAG AGA CAG TCC AAC GGC GTA GTT GAA CAT TTT GTT GCA TAG GCC ACT AGT GGA TCT G-3'<br>5'-CTA GGC CAT CGT ACA CTA TAG C-3'<br>5'-GCG TAC GAA GCT TCA GCT G-3'	pUG6-kanMX (Gueldener et al., 2002)
Deletion of *CCC1* in WT (1) or in Δ*smf3* (2)	5'-CAA AGA AAC TTT TTT TTT GTC CTC CCA TAT CTC GTG CAC ACA AAT ATT ATG CGT ACG CTG CAG GTC GAC-3'<br>5'-CCA AAT TTC TTT ACA ATA AAT ATG TAT AGT GTT GTT ACT TTA CAC TTA ATC GAT GAA TTC GAG CTC G-3'<br>5'-GCC AAC AAT GGT CAC TTC GAC-3'<br>5'-GTC GAC CTG CAG CGT ACG-3'	pFA6a-natNT2 (1) or pFA6a-hphNT1 (2) (Janke et al., 2004)
Deletion of *SMF3*	5'-CTT CCA AGC TAA TTG ATA ACA GTA GTA GCA TCA CAA GAG AAA AAA AAT GCG TAC GCT GCA GGT CGA C-3'<br>5'-CAA GAA TCG AGA GAA AAC GAC ACA CTT GAC AAT AAA TAG GTT CCA TTA ATC GAT GAA TTC GAG CTC G-3'<br>5'-CTC TCT TCC AGT TTC CGC AC-3'<br>5'-GTC GAC CTG CAG CGT ACG-3'	pFA6a-natNT2 (Janke et al., 2004)

Deletion of *YPK9* in WT (3), in Δ*smf3*Δ*ccc1* (4) or in Δ*ccc1* and Δ*smf3* (5)	5'-TAA AAT AAA AAG GAG CCC AGA CTT ACT GAT AGA TCT TGC ATA TAC TCC GGT AAT GCG TAC GCT GCA GGT CGA C-3'<br>5'-TTT AAT TAT AGA ACA TGG TAC TTG TAC ACA TAC ATA GAT AAA AAT CTT TGC TTT AAT CGA TGA ATT CGA GCT CG-3'<br>5'-GTC CTA CCG CCT CAG GAT G-3'<br>5'-GTC GAC CTG CAG CGT ACG-3'	pFA6a-natNT2 (3), pFA6a-hphNT1 (4) (Janke et al., 2004) or pFA6a-kanMX (This study)
Deletion of *GDT1*	5'-GTT AGG TTC TAC AAA GCA AGA TAC AAA TAC GAG GCC ATC GAG TAT TCA ATG CGT ACG CTG CAG GTC GAC-3'<br>5'-GAA GAG AAA AAA GAG AGA ATC CAA GAA GAA TAC GGT TAC AAA CCA TCT CAA TCG ATG AAT TCG AGC TCG-3'<br>5'-GCG ATC AAT ATA GAC CGT TG-3'<br>5'-GTC GAC CTG CAG CGT ACG-3'	pFA6a-natNT2 (Janke et al., 2004)
Deletion of *SPF1*	5'-GAC ATA GTT GAC ATA TCA GAC CTA CAG AAA CAT AGG AAT CGG TAA ATG CGT ACG CTG CAG GTC GAC-3'<br>5'-GTA ATA TAA GTA TAT AAA TAC AAA AAG GGG TAC TAC ATA AAA GAT TTA TCA ATC GAT GAA TTC GAG CTCG-3'<br>5'-CAG GAA TGT TTA CTA AAA GAC-3'<br>5'-GTC GAC CTG CAG CGT ACG-3'	pFA6a-natNT2 (Janke et al., 2004)
Deletion of *COQ7*	5'-CAA GGA ATA AAG ATA TCA CGT ATA CGG GAG AGA TAC ATA GAA ATT ATG CGT ACG CTG CAG GTCGAC-3'<br>5'-TTT TCT GGC ATA ACG CGA CTG ATG TAT GCC ACT TTC TGG TGG TTA ATC GAT GAA TTC GAG CTCG-3'<br>5'-CGT ACT CTG TCT ATA TTT CCC-3'<br>5'-GTC GAC CTG CAG CGT ACG-3'	pFA6a-natNT2 (Janke et al., 2004)
C-terminal tagging of Coq1	5'-CTT CAT TTT CTC TTT ATT CTT CAC CCT TTA TTT GAA ATT TCA AGG TAT CGA TGA ATT CGA GCT CG-3'<br>5'-GTT CTG CCC TAG AGT TTT TAA CTA ATA GTA TAC TAA CAA GAA GAA AGC GTA CGC TGC AGG TCG AC-3'<br>5'-CCA TAA TGG TAT AGC GAA GAC G-3'<br>5'-CCG TAA GTA GCA TCA CCT TC-3'	pYM25 (Janke et al., 2004)
C-terminal tagging of Coq9	5'-CTA CGC ATT TCA CTG TCC TAA ACT TCA AAT TGA CAT GTA AGA TTG CAT CGA TGA ATT CGA GCT CG-3'<br>5'-GTT AAT GTC TAC GGT AAA TTT AAT CAA ATC TCA ATT AGT TAG GGG TCG TAC GCT GCA GGT CGA C-3'<br>5'-ATG CTA AGA GAT TGG CTG TTT C-3'<br>5'-CCG TAA GTA GCA TCA CCT TC-3'	pYM25 (Janke et al., 2004)
PIM1 promoter exchange	5'-GTG GTC CTC GCC ACT GTG CTA AGG GTC TTT GTG GTT TTG TTC TTA GCA TCG ATG AAT TCT CTG TCG-3'<br>5'-GTT TTT TCT TTT GGT TTT CGA GGT GCT TGA ACG AAA GAT TTG CAA ATA GAG CAT GCG TAC GCT GCA GGT CGA C-3'<br>5'-CGA AGA GGT GCC TTG AGA AG-3'<br>5'-GTC GAC CTG CAG CGT ACG-3'	pYM-HIS-N-tetOFF (This sudy)
**Delitto perfetto**	**Oligonucleotides**	**Template**
Insertion of counter selection cassette replacing *PMR1* codon 53	5'-TAG AGT ATT GTA CTT TAT CCG TGG ACG AAG CTC TAG AAA AAC TGG ACA CTG AGC TCG TTT TCG ACA CTG G-3'<br>5'-AGT GAT CTC CTA TTG TTG GCC TCG TTA GAT GAT CGT AAA CCA CCG TTT TTT CCT TAC CAT TAA GTT GAT C-3'	pCORE (Storici and Resnick, 2006)
Colony PCR to verify insertion	5'-AGT CGT CAC TCA TGG TGA TTT C-3'<br>5'-CTA GGC CAT CGT ACA CTA TAG C-3'	

Introduction of point mutation Pmr1^{D53A}, replacing *PMR1* codon 53 (GAC->GCC)	5'-CTT TAT CCG TGG ACG AAG CTC TAG AAA AAC TGG ACA CTG CCA AAA ACG GTG GTT TAC GAT CAT CTA ACG AGG CCA ACA A-3'<br>5'-GAA ATA GGC ACC TGC TTC GAG ATC TTT TTG ACC TGT GAC GGT TTT TGC CAC CAA ATG CTA GTA GAT TGC TCC GGT TGT T-3'	
Sequencing	5'-GCA CAG ACG TAA GCT TAA G-3'<br>5'-GAT CTC TCA GAA ATC GG-3'	
Insertion of counter selection cassette replacing *PMR1* codon 783	5'-ACG CAA TGC AAA TTC TTT GGA TAA ATA TTT TAA TGG ATG GGC CAC CAG CTG AGC TCG TTT TCG ACA CTG G-3'<br>5'-CTT GGA GGT TTT TTC ATA ACT TCA TGA TCA ACA GGT TCC ACA CCT AAG GAT CCT TAC CAT TAA GTT GAT C-3'	pCORE (Storici and Resnick, 2006)
Colony PCR to verify insertion	5'-AGA CGA CAA AGG CGA TGC ATT G-3'<br>5'-GCT TTG TAT ATA CGA AAA GTC GCC-3'	
Introduction of point mutation Pmr1^{Q783A}, replacing *PMR1* codon 783 (CAA->GCA)	5'-GCA AAT TCT TTG GAT AAA TAT TTT AAT GGA TGG GCC ACC AGC TGC ATC CTT AGG TGT GGA ACC TGT TGA TCA TGA AGT TAT G-3'<br>5'-CGT TTA AGA AAC CTA TTT ATA AAA TTA CCT ACC CGG TGG TCG ACG TAG GAA TCC ACA CCT TGG ACA ACT AGT ACT TCA ATA C-3'	
Sequencing	5'-GAA GCC TCA GAT ATG GTC-3'<br>5'-CGT TGA GTC TTC TTC ATT C-3'	
Insertion of counter selection cassette for ^{GFP}Coq7	5'-CTT TAA AGA TAA CAG AAC ATA CAT CAG CAA AAC ACA CCG AAA AAC CTG AGG AGC TCG TTT TCG ACA CTG G-3'<br>5'-TCC AAA AAT GCA GCC TGA GCA TCT GAT AAA TTC TGA CAC TTG GGA GCA TGT CCT TAC CAT TAA GTT GAT C-3'	pCORE (Storici and Resnick, 2006)
Colony PCR to verify insertion	5'-AGT CGT CAC TCA TGG TGA TTT C-3'<br>5'-CGT ACT CTG TCT ATA TTT CCC-3'	
Amplification of GFP for tagging Coq7 between amino acid 36 and 37	5'-CTT TAA AGA TAA CAG AAC ATA CAT CAG CAA AAC ACA CCG AAA AAC CTG AGT CTA AAG GTG AAG AAT TAT TCA C-3'<br>5'-TCC AAA AAT GCA GCC TGA GCA TCT GAT AAA TTC TGA CAC TTG GGA GCA TGA CGC GTG GTG GAG GAG GTT CTG GTG GCG GTG GAT CTG TAC AAT TCA TCC ATA CC-3'	pYM-N9 (Janke et al., 2004)
Sequencing	5'-CGT ACT CTG TCT ATA TTT CCC-3'<br>5'-CCT TAG ACT ATG AAC CCA TAC-3'	

Supplementary Table 6. Oligonucleotides used in this study to construct plasmids.

Purpose	Enzyme	Oligonucleotides	Template
Amplification of *COQ4* + 184 bp upstream of start codon and restriction cloning into pHR81-URA-VC	SacI<br>BamHI	5'-ATC TGA GCT CGC TTT TCG AGC ACT TCC ACT G-3'<br>5'-ATC TGG ATC CTC ATG CTG GAG TCG TGG CTC -3'	BY4741 genomic DNA
Sequencing		5'-GTC TCA TCC TTC AAT GCT ATC A-3'<br>5'-TGT AAA ACG ACG GCC AGT-3'	
Amplification of *COQ4* + 500 bp upstream of start codon and restriction cloning into pHR81-URA-VC	SacI<br>BamHI	5'-ATC TGA GCT CCC CCT TTG AAA TTG TGG GAA TC-3'<br>5'-ATC TGG ATC CTC ATG CTG GAG TCG TGG CTC -3'	BY4741 genomic DNA
Sequencing		5'-GTC TCA TCC TTC AAT GCT ATC A-3'<br>5'-TGT AAA ACG ACG GCC AGT-3'	
Amplification of *COQ7* + 500 bp upstream of start codon and restriction cloning into pHR81-URA-VC	SacI<br>BamHI	5'-ATC TGA GCT CCA TTT ATT CCG ATG CCT TAG -3'<br>5'-ACT GGA TCC TTA AAT TCT TTC GGC ACT CC- 3'	pPM6
Sequencing		5'-GTC TCA TCC TTC AAT GCT ATC A-3'<br>5'-TGT AAA ACG ACG GCC AGT-3'	
Amplification of *COQ8* + 500 bp upstream of start codon and homologous recombination in *S. cerevisiae* after linearizing plasmid with SacI and BamHI		5'-TCC CAG TCA CGA CGT TGT AAA ACG ACG GCC AGT GAA TTC GAT TCT TGC AGT CCT AAT CTT-3'<br>5'-TTT GAT ATT GGA TCA TAT GCA TAG TAC CGA GAA ACT AGA GTT AAA CTT TAT AGG CAA AAA TC-3'	BY4741 genomic DNA
Sequencing		5'-GTC TCA TCC TTC AAT GCT ATC A-3'<br>5'-TGT AAA ACG ACG GCC AGT-3'	
Amplification of *COQ9* + 500 bp upstream of start codon and cloning into pHR81-URA-VC or pHR-HIS-VC	BamHI<br>BamHI	5'-GAA TTG GAT CCG ATG TCT TAA TGG AGC CCG TC-3'<br>5'-GCA CTG GAT CCT TAA CCC CTA ACT AAT TGA GATTTG-3'	BY4741 genomic DNA
Sequencing		5'-GTC TCA TCC TTC AAT GCT ATC A-3'<br>5'-TGT AAA ACG ACG GCC AGT-3'	
Amplification of *IAH1* + 500 bp upstream of start codon and restriction cloning into pHR81-URA-VC	SacI<br>BamHI	5'-ATC TGA GCT CGA TGC CAT TTT AAT ATG CCG -3'<br>5'-ACT GGA TCC TCA AGA CAT TAT GTT AGA TCC -3'	BY4741 genomic DNA
Sequencing		5'-GTC TCA TCC TTC AAT GCT ATC A-3'<br>5'-TGT AAA ACG ACG GCC AGT-3'	
Amplification of *COQ7* and restriction cloning into pCM190-URA-VC	BamHI<br>NotI	5'-ATC TGG ATC CCC CGC CGC CAC CAT GTT ATC CCG TGT TTC AGT TTT CA-3'<br>5'-ATC TGC GGC CGC TTA AAT TCT TTC GGC ACT CCA TAT A-3'	BY4741 genomic DNA
Sequencing		5'-CTA AAT ATT CTT TCC TTA TAC ATT AG-3'<br>5'-CAG GAA ACA GCT ATG ACC-3'	
Amplification of *COQ7* and restriction cloning into pUG35-URA-VC	SpeI<br>ClaI	5'-ATC TAC TAG TAT GTT ATC CCG TGT TTC AGT TTT CA-3'<br>5'-ATC TAT CGA TTT AAA TTC TTT CGG CAC TCC ATA TA-3'	BY4741 genomic DNA
Sequencing		5'-CAG GAA ACA GCT ATG ACC-3'<br>5'-CCG TAA GTA GCA TCA CCT TC-3'	

Amplification of Fragment 1 (F1) and F2 for generation of FLAGCoq7 via homologous recombination in *S. cerevisiae* after linearizing pHR81-URA-VC with SacI and BamHI	F1	5'-CAG TCA CGA CGT TGT AAA ACG ACG GCC AGT GAA TTC GAG CTC CAT TTA TTC CGA TGC CTT AG-3'<br>5'-CGC CAC CAG AAC CTC CTC CAC CAC GCG TTT TAT CAT CAT CAT CTT TAT AAT CCT CAG GTT TTT CGG TGT G-3'	pHR81-COQ7
	F2	5'-GAT GAT GAT GAT AAA ACG CGT GGT GGA GGA GGT TCT GGT GGC GGT GGA TCT CAT GCT CCC AAG TGT CAG -3'<br>5'-CAT TTC CTT TGA TAT TGG ATC ATA TGC ATA GTA CCG AGA AAC TAG AGG ATC CTT AAA TTC TTT CGG CAC TCC-3'	pHR81-COQ7
Sequencing		5'-CGT ACT CTG TCT ATA TTT CCC-3'<br>5'-CAT CCT TCA ATG CTA TCA-3'	
Amplification of *HIS3* for marker exchange and homologous recombination in *S. cerevisiae* after linearizing plasmid with HindIII	HindIII	5'-CAG TAG ACG GAG TAT ACT AGA GTC GAC CTG CAG GCA TGC AAG CTT CTA TTA CTC TTG GCC TCC TCT AG-3'<br>5'-CAA TTT CAC ACA GGA AAC AGC TAT GAC CAT GAT TAC GCC AAG CTT CAC CGC ATA GAT CCG TCG AG-3'	BY4741 genomic DNA
Amplification of *PMR1* and restriction cloning into pRS313-HIS-VC	SacI	5'-ATC TGA GCT CTC AAA CAT TTG AGA AAT ACG TTG AGT C-3'<br>5'-ATC TGA GCT CAC GTT TCA CAG GCA TAT TTG AC-3'	BY4741 genomic DNA
Amplification of tetO7-CYC1 promoter (incl. tTA domain) and restriction cloning into pFA6a-His3MX6	EcoRI	5’-GTC GAT TCG ATA CTA ACG CCG CCA TCC AGT GTC GAA AAC GAG CTC ATA TGT AAA ACG ACG GCC AGT GAA TTC TTA TTA CGA TCC TCG CGC-3’<br>5’-CAT AGG CCA CTA GTG GAT CTG ATA TCA TCG ATG AAT TCT CTG TCG GGA TCC CCC GAA TTG ATC CGG TAA TTT AGT GTG TGT ATT TGT G-3’	pCM190-URA-VC

Supplementary Table 7. Instrument specifications S2 Picofox spectrometer

**X-ray tube**	Molybdenum, max. 50 W (max. 50 kV, max. 1 mA)
**Detector, area, resolution**	XFlash 430 Picofox, Be / 30 mm^2<br>Energy resolution ≤ 150 eV (Kα-Mn)
**Carrier**	Quartz (ø 30 mm)
**Sample station**	Automatic (cassette for 25 carriers)
**Control and software**	PC, Spectra v7.8.2
**Gain correction**	1 µg As on quartz carrier: daily
**Spectroscopic resolution test**	1 µg Mn on quartz carrier: 132 eV
**Sensitivity test**	1 ng Ni on quartz carrier<br>Measured value sensitivity: 46 counts ng^{-1} mA^{-1} s^{-1}<br>Measured value (Lowest limit of detection): 0.104 pg
**Quantification test**	3 sample carrier with 1 µl Kraft 13:<br>All measured values (Ti, V, Cr, Mn, Fe, Co, (Ni) Cu, Zn, Rb)<br>within target values (Ni was set as internal standard)
**Manufacturer**	Bruker Nano GmbH, Germany

Supplementary Table 8. Oligonucleotides used in this study for qRT-PCR.

Gene	Oligonucleotides
COQ1	5'-GCG GAG GAA TAT AGG GAC AAG G-3'<br>5'-AAC TCT AGG GCA GAA CGA GC-3'
COQ4	5'-GCG AAA GAA GCT GAA GAG CG-3'<br>5'-TGT ACC GAG GTT CCA TTG GC-3'
COQ5	5'-GCC AGA GCA GAA GTA TAC GCA-3'<br>5'-CGA TTG GCC ACG GAA GAA AAG-3'
COQ6	5'-CAC AGC GAC CCT AAT GAT CCA-3'<br>5'-TGC ACC CAC TAG CAA TCT CG-3'
COQ7	5'-GCA TGC TCC CAA GTG TCA GA-3'<br>5'-CGC CAG CTT GAT CTA CAC GA-3'
COQ9	5'-CAA CCC TGA CGT AAC TCC ACA-3'<br>5'-CGA CAA ATG CCC ACC GAT TG-3'
UBC6	5'-GGA CGT TTC AAG CCC AAC AC-3'<br>5'-TGA GAC AGA CCA GCC AGG AT-3'

Supplementary Table 9. Details of statistical analyses performed

Analysed Data	Statistical test	Additional information	Result
**Figure 1**			
(h) O_2 consumption	Two-sided Wilcoxon rank sum test	Outlier(s) outside of 1.5-fold IQR detected	WT vs Δ*pmr1*<br>W = 121, p-value = 2.835e-06
(j) Mn levels	Kruskal-Wallis rank sum test with pairwise comparison using Games-Howell test	Outlier(s) outside of 1.5-fold IQR detected	chi-squared = 25.706, df = 3, p-value = 1.099e-05<br>WT vs $Pmr1^{Q783A}$: p = 7.8e-06<br>WT vs Δ*pmr1*: p = 2.9e-09
(l) Mn levels	Kruskal-Wallis rank sum test with pairwise comparison using Games-Howell test	Outlier(s) outside of 1.5-fold IQR detected	chi-squared = 20.48, df = 2, p-value = 3.571e-05<br>ctrl vs 5 mM Mn: p = 2.2e-08<br>ctrl vs 10 mM Mn: p = 5.9e-08
(m) O_2 consumption	One-way analysis of variance with Bonferroni post-hoc test	All assumptions met	F(2) = 157.22, p < 2.721e-16<br>ctrl vs 5 mM Mn: p = 0.00029<br>ctrl vs 10 mM Mn: p < 2e-16
**Figure 2**			
(e) CoQ levels	One-way analysis of variance with Bonferroni post-hoc test	All assumptions met	F(3) = 94.69, p < 1.277e-08<br>WT vs $Pmr1^{D53A}$: p = 1<br>WT vs $Pmr1^{Q783A}$: p = 2.1e-07<br>WT vs Δ*pmr1*: p = 1.4e-07
(f) DMQ levels	Kruskal-Wallis rank sum test with pairwise comparison using Games-Howell test	Outlier(s) outside of 1.5-fold IQR detected	chi-squared = 12, df = 3, p-value = 0.007383<br>WT vs $Pmr1^{D53A}$: p = 0.3387<br>WT vs $Pmr1^{Q783A}$: p = 0.0190<br>WT vs Δ*pmr1*: p = 0.0090
(h) CoQ levels	Kruskal-Wallis rank sum test with pairwise comparison using Games-Howell test	Outlier(s) outside of 1.5-fold IQR detected	chi-squared = 9.8462, df = 2, p-value = 0.007277<br>ctrl vs 5 mM Mn: p = 0.0013<br>ctrl vs 10 mM Mn: p = 6e-05
(i) DMQ levels	Kruskal-Wallis rank sum test with pairwise comparison using Games-Howell test	Outlier(s) outside of 1.5-fold IQR detected	chi-squared = 9.8462, df = 2, p-value = 0.007277<br>ctrl vs 5 mM Mn: p = 2.0e-05<br>ctrl vs 10 mM Mn: p = 7.9e-06
**Figure 3**			
(a) O_2 consumption	One-way analysis of means (not assuming equal variances) with pairwise comparison using Games-Howell test	Non-homogeneous variances in data (Levene's Test, p-value = 0.005444)	F = 479.02, num df = 3.000, denom df = 12.417, p-value = 4.346e-13<br>Δ*pmr1* VC vs Δ*pmr1* Coq7: p = 2.9e-07
(d) O_2 consumption	Kruskal-Wallis rank sum test with pairwise comparison using Games-Howell test	Outlier(s) outside of 1.5-fold IQR detected	chi-squared = 20.406, df = 3, p-value = 0.0001398<br>VC ctrl vs VC 5 mM Mn: p = 4.9e-05<br>VC 5 mM Mn vs Coq7 5 mM Mn: p = 0.0023

(f) CoQ levels	Kruskal-Wallis rank sum test with pairwise comparison using Games-Howell test	Outlier(s) outside of 1.5-fold IQR detected	chi-squared = 14.118, df = 3, p-value = 0.002749<br>Δ*pmr1* VC vs Δ*pmr1* Coq7: p < 2e-16
(h) CoQ levels	Kruskal-Wallis rank sum test with pairwise comparison using Games-Howell test	Outlier(s) outside of 1.5-fold IQR detected	chi-squared = 14.118, df = 3, p-value = 0.002749<br>WT 5 mM Mn VC vs WT 5 mM Mn Coq7: p = 1.5e-05
**Figure 4**			
(c) CIV activity	Two-sided Two Sample t-test	All assumptions met	WT vs Δ*pmr1*<br>t = -5.5603, df = 4, p-value = 0.005122
(d) CIII activity	Two-sided Two Sample t-test	All assumptions met	WT vs Δ*pmr1*<br>t = -1.6543, df = 4, p-value = 0.1734
(e) CIV activity	Two-sided Two Sample t-test	All assumptions met	WT ctrl vs WT Mn<br>t = -1.603, df = 4, p-value = 0.1842
(f) CIII activity	Two-sided Two Sample t-test	All assumptions met	WT ctrl vs WT Mn<br>t = 1.3694, df = 4, p-value = 0.2427
(g) Succinate-CIII activity ± dCoQ	One-way analysis of variance with Bonferroni post-hoc test	All assumptions met	F(3) = 14.582, p = 0.001316<br>WT (-) vs Δ*pmr1* (-): p = 0.0081<br>Δ*pmr1* (-) vs Δ*pmr1* (+): p = 0.0017
(h) NADH-CIII activity ± dCoQ	One-way analysis of variance with Bonferroni post-hoc test	All assumptions met	F(3) = 60.108, p = 7.876e-06<br>WT (-) vs Δ*pmr1* (-): p = 1.4e-05<br>Δ*pmr1* (-) vs Δ*pmr1* (+): p = 5.3e-05
(i) NADH-CIII activity ± dCoQ	One-way analysis of variance with Bonferroni post-hoc test	Due to equal sample size in all groups, test was conducted despite non-normally distributed data (Shapiro-Wilk, p = 0.041)	F(3) = 42.368, p = 2.953e-05<br>WT ctrl vs WT Mn: p = 3.1e-05<br>WT Mn vs WT Mn dCoQ: p = 0.00713
(j) NADH-CIII activity	One-way analysis of variance with Bonferroni post-hoc test	All assumptions met	F(3) = 20.836, p = 0.0003889<br>Δ*pmr1* VC vs Δ*pmr1* Coq7: p = 0.00066
**Figure 5**			
(f) Coq7 protein levels	One-way analysis of variance with Bonferroni post-hoc test	All assumptions met	F(3) = 80.163, p = 3.31e-08<br>Δ*pmr1* $Pim1^{ON}$ vs $Pim1^{OFF}$: p = 6.4e-06
(i) Mn/Fe	Kruskal-Wallis rank sum test with pairwise comparison using Games-Howell test	Outlier(s) outside of 1.5-fold IQR detected	chi-squared = 22.934, df = 2, p-value = 1.047e-05<br>WT ctrl vs Δ*pmr1*: p = 0.0011<br>WT ctrl vs Mn: p = 0.0023
(j) Coq7 activity	One-way analysis of variance with Bonferroni post-hoc test	All assumptions met	F(2) = 17.412, p = 0.003175<br>WT ctrl vs Δ*pmr1*: p = 0.0264<br>WT ctrl vs Mn: p = 0.0034

**Figure 6**			
(a) Mn levels larva	Two-sided Wilcoxon rank sum test	Outlier(s) outside of 1.5-fold IQR detected	w1118 (n=7) vs SPoCk (n=7)<br>W = 9, p-value = 0.05303
(b) Mn levels adult	Two-sided Two Sample t-test	All assumptions met	w1118 (n=4) vs SPoCk (n=4)<br>t = -4.2646, df = 6, p-value = 0.005294
(d) Mitotracker CMXRos	Two-sided Two Sample t-test	All assumptions met	w1118 (n=10) vs SPoCk (n=9)<br>t = 2.3439, df = 17, p-value = 0.03149
(e) CoQ levels larval muscle	Two-sided Two Sample t-test	All assumptions met	w1118 CoQ_9 (n=9) vs SPoCk CoQ_9 (n=9)<br>t = 2.5063, df = 16, p = 0.02338
(f) CoQ levels thorax muscle	Two-sided Two Sample t-test	All assumptions met	w1118 CoQ_9 (n=4) vs SPoCk CoQ_9 (n=4)<br>t = 4.3144, df = 6, p = 0.005013
(g) Eclosed flies	Log-rank (Mantel-Cox) test with Bonferroni post-hoc test	Comparisons were conducted between the following groups:	
		w1118 ctrl (n=274)	w1118 ctrl vs SPoCk CoQ_{10} p = 0.00003
		SPoCk ctrl (n=232)	w1118 ctrl vs w1118 CoQ_{10} p = 0.202848
		w1118 CoQ_{10} (n=246)	SPoCk ctrl vs SPoCk CoQ_{10} p = 0.000012
		SPoCk CoQ_{10} (n=208)	w1118 CoQ_{10} vs SPoCk CoQ_{10} p = 1.491102
(h) Eclosed flies	Log-rank (Mantel-Cox) test with Bonferroni post-hoc test	Comparisons were conducted between the groups:	
		w1118 ctrl (n=335)	w1118 ctrl vs SPoCk ctrl p = 0.000000144
		SPoCk ctrl (n=293)	w1118 ctrl vs w1118 diHB p = 5.845278
		w1118 diHB (n=295)	SPoCk ctrl vs SPoCk diHB p = 0.000216
		SPoCk diHB (n=232)	w1118 diHB vs SPoCk diHB p = 2.271558
(i) Dead flies	One-way analysis of variance with Bonferroni post-hoc test	Conducted test despite non-normally distributed data (Shapiro-Wilk, p = 0.00466) and non-homogeneous variance (Levene's Test, p = 1.253e-09), as value for w1118 diHB group = 0 and cannot have any variation. Data of SPoCk ctrl and diHB groups are normally distributed.	F(3) = 67.444, p < 2.2e-16<br>w1118 ctrl (n=16) vs SPoCk ctrl (n=16): p = 2.8e-15<br>SPoCk ctrl (n=16) vs SPoCk diHB (n=13): p = 0.027

**Supplementary Figure 1**			
(c) Mitotracker DeepRed	Two-sided Two Sample t-test	All assumptions met	t = 8.3668, df = 14<br>p-value = 8.096e-07
(d) Mn levels	Kruskal-Wallis rank sum test with pairwise comparison using Games-Howell test	Outlier(s) outside of 1.5-fold IQR detected	chi-squared = 13.097, df = 3, p-value = 0.004431<br>Δ*pmr1* VC vs Δ*pmr1* Pmr1: p = 7.6e-05
(f) Cell death 24 h	Kruskal-Wallis rank sum test with pairwise comparison using Games-Howell test	Outlier(s) outside of 1.5-fold IQR detected	chi-squared = 12.199, df = 3, p-value = 0.006733<br>Δ*pmr1* VC vs Δ*pmr1* Pmr1: p = 0.018
(f) Cell death 48 h	One-way analysis of means (not assuming equal variances) with pairwise comparison using Games-Howell test	Non-homogeneous variances in data (Levene's Test, p-value = 0.0001071)	F = 15.483, num df = 3.0000, denom df = 6.3936, p-value = 0.002499<br>Δ*pmr1* VC vs Δ*pmr1* Pmr1: p = 0.017
**Supplementary Figure 4**			
(a) COQ mRNA levels WT vs Δ*pmr1*	Two-sided Wilcoxon rank sum test	Outlier(s) outside of 1.5-fold IQR detected	COQ1: W = 5, p-value = 0.04113
	Two-sided Wilcoxon rank sum test	Outlier(s) outside of 1.5-fold IQR detected	COQ4: W = 5, p-value = 0.04495
	Two-sided Wilcoxon rank sum test	Outlier(s) outside of 1.5-fold IQR detected	COQ5: W = 0, p-value = 0.002165
	Two-sided Welch Two Sample t-test	Non-homogeneous variances in data (Levene's Test, p-value = 0.004787)	COQ6: t = -6.9066, df = 5.4165, p-value = 0.0007027
	Two-sided Wilcoxon rank sum test	Outlier(s) outside of 1.5-fold IQR detected	COQ7: W = 34, p-value = 0.8785
	Two-sided Two Sample t-test	All assumptions met	COQ9: t = -4.9126, df = 10, p-value = 0.0006115
(d) Coq7 protein levels	Kruskal-Wallis rank sum test with pairwise comparison using Games-Howell test	Outlier(s) outside of 1.5-fold IQR detected	chi-squared = 18.514, df = 3, p-value = 0.0003445<br>WT VC vs WT Coq7: p = 0.0044<br>Δ*pmr1* VC vs Δ*pmr1* Coq7: p = 0.0642
(e) Coq7 mRNA levels	Kruskal-Wallis rank sum test with pairwise comparison using Games-Howell test	Outlier(s) outside of 1.5-fold IQR detected	chi-squared = 18.187, df = 3, p-value = 0.0004025<br>WT VC vs WT Coq7: p = 0.0048<br>Δ*pmr1* VC vs Δ*pmr1* Coq7: p = 0.0034
