# Supplementary material for: Manganese-driven CoQ deficiency
Source: Nat Commun. 2022 Oct 13;13:6061. doi: 10.1038/s41467-022-33641-x (PMC9563070; doi:10.1038/s41467-022-33641-x)
Supplement: Supplementary file 3 — Description of Additional Supplementary Files [file 41467_2022_33641_MOESM3_ESM.pdf]

Inventory of Supporting Information

Supplementary Information.

Including:

Supplementary Figure 1. Cellular Mn overload results in accumulation of Mn within mitochondria, reduced mitochondrial mass and extra-mitochondrial accumulation of reactive oxygen species.
Supplementary Figure 2. Overexpression of Coq4 does not prevent Mn-driven respiratory defects.
Supplementary Figure 3. Proteins associated with CoQ metabolism are not generally deregulated upon Mn overload.
Supplementary Figure 4. Mn overload causes destabilization of Coq7.
Supplementary Figure 5. Gating strategies for flow cytometry.
Supplementary Table 1. Total cellular Mn levels in yeast mutants lacking proteins involved in Mn homeostasis.
Supplementary Table 2. Total cellular metal concentrations in cells lacking Pmr1.
Supplementary Table 3. Yeast strains used in this study.
Supplementary Table 4. Plasmids used in this study.
Supplementary Table 5. Oligonucleotides used for gene deletion, chromosomal tagging and introduction of point mutations.
Supplementary Table 6. Oligonucleotides used to construct plasmids.
Supplementary Table 7. Instrument specifications S2 Picofox spectrometer
Supplementary Table 8. Oligonucleotides used in this study for qRT-PCR.
Supplementary Table 9. Details of statistical analyses performed.

Supplementary Data 1.

DeqMS analysis of proteomics data, corresponding to Fig. 1e.

Reporting Summary.

Source Data file.

Contains numerical data, gels and blots for all graphs presented within the Figures and Supplementary Figures.
