# Supplementary material for: Manganese-driven CoQ deficiency
Source: Nat Commun. 2022 Oct 13;13:6061. doi: 10.1038/s41467-022-33641-x (PMC9563070; doi:10.1038/s41467-022-33641-x)
Supplement: Supplementary file 5 — Reporting Summary [file 41467_2022_33641_MOESM5_ESM.pdf]

nature portfolio

Corresponding author(s): Sabrina Büttner

Last updated by author(s): Sep 22, 2022

Reporting Summary

Nature Portfolio wishes to improve the reproducibility of the work that we publish. This form provides structure for consistency and transparency in reporting. For further information on Nature Portfolio policies, see our Editorial Policies and the Editorial Policy Checklist.

Statistics

For all statistical analyses, confirm that the following items are present in the figure legend, table legend, main text, or Methods section.

n/a	Confirmed	
☐	☒	The exact sample size (n) for each experimental group/condition, given as a discrete number and unit of measurement
☐	☒	A statement on whether measurements were taken from distinct samples or whether the same sample was measured repeatedly
☐	☒	The statistical test(s) used AND whether they are one- or two-sided<br>*Only common tests should be described solely by name; describe more complex techniques in the Methods section.*
☒	☐	A description of all covariates tested
☐	☒	A description of any assumptions or corrections, such as tests of normality and adjustment for multiple comparisons
☐	☒	A full description of the statistical parameters including central tendency (e.g. means) or other basic estimates (e.g. regression coefficient) AND variation (e.g. standard deviation) or associated estimates of uncertainty (e.g. confidence intervals)
☐	☒	For null hypothesis testing, the test statistic (e.g. F, t, r) with confidence intervals, effect sizes, degrees of freedom and P value noted<br>*Give P values as exact values whenever suitable.*
☒	☐	For Bayesian analysis, information on the choice of priors and Markov chain Monte Carlo settings
☒	☐	For hierarchical and complex designs, identification of the appropriate level for tests and full reporting of outcomes
☒	☐	Estimates of effect sizes (e.g. Cohen's d, Pearson's r), indicating how they were calculated

Our web collection on statistics for biologists contains articles on many of the points above.

Software and code

Policy information about availability of computer code

Data collection	For data collection the following softwares and code were used: Spectra v7.8.2, Proteome Discoverer vs. 1.4 including Sequest-Percolator, LabChart v7.3.8, InCyte v3.1, ZEN blue edition v2.3.69.1018 and ZEN 2011 black edition v7.0.5.288.
Data analysis	Data were analyzed and graphs were generated with GraphPad Prism v8.0, Fiji v1.53q, Adobe Illustrator v26.5 and RStudio v1.2.5 with the following packages and functions: base, DeqMS, ggplot2, gplots, tidyverse, car::leveneTest, stats::kruskal.test, stats::oneway.test, stats::pairwise.t, stats::prcomp, stats::shapiro.wilk, stats::t.test, stats::wilcox.test, PMCMRplus::gamesHowellTest.

For manuscripts utilizing custom algorithms or software that are central to the research but not yet described in published literature, software must be made available to editors and reviewers. We strongly encourage code deposition in a community repository (e.g. GitHub). See the Nature Portfolio guidelines for submitting code & software for further information.

Data

Policy information about availability of data

All manuscripts must include a data availability statement. This statement should provide the following information, where applicable:

- Accession codes, unique identifiers, or web links for publicly available datasets
- A description of any restrictions on data availability
- For clinical datasets or third party data, please ensure that the statement adheres to our policy

A Data Availability Statement is provided. All source data are provided with this paper. The mass spectrometry proteomics data have been deposited to the ProteomeXchange Consortium via the PRIDE partner repository (https://www.ebi.ac.uk/pride) with the dataset identifier PXD036952. In addition, the DeqMS analysis of proteomics data is available in Supplementary Data 1, and corresponding raw data is provided in the Source Data file.

March 2021

Field-specific reporting

Please select the one below that is the best fit for your research. If you are not sure, read the appropriate sections before making your selection.

☒ Life sciences ☐ Behavioural & social sciences ☐ Ecological, evolutionary & environmental sciences

For a reference copy of the document with all sections, see nature.com/documents/nr-reporting-summary-flat.pdf

Life sciences study design

All studies must disclose on these points even when the disclosure is negative.

Sample size	No statistical methods were used to predetermine sample size. Experiments were performed on at least 3 biologically independent samples as per commonly accepted field standards and to enable statistical analysis (e.g. calculation of mean and SEM). For yeast experiments, 1 n represents cells harvested from one flask. For Mn quantification in Drosophila, 1 n represents 10 third instar larvae or 5 whole flies, and for CoQ quantification in Drosophila, 1 n represents 10 inverted third instar larvae or 10 thoraxes from adult flies. For microscopic analysis of mitochondrial transmembrane potential in Drosophila larval muscle tissue, 1 n represents one animal.
Data exclusions	No samples or data points were excluded from the analysis.
Replication	All data were obtained from at least 3 independent experiments, and exact numbers of independent experiments are listed in the respective figure legends. All attempts at replication were successful.
Randomization	Randomization is not applicable in applied experimental settings, and no group allocation was performed. Experiments were performed comparing either different mutant strains or treatments on otherwise isogenic samples.
Blinding	Not relevant to this study, as no group allocation was used. Each investigator performing a given experiment labeled the corresponding samples and performed the analysis. Appropriate cellular and biochemical controls were included in each experimental replication and results reported are predominantly quantitative in nature.

Reporting for specific materials, systems and methods

We require information from authors about some types of materials, experimental systems and methods used in many studies. Here, indicate whether each material, system or method listed is relevant to your study. If you are not sure if a list item applies to your research, read the appropriate section before selecting a response.

Materials & experimental systems

n/a	Involved in the study	
☐	☒	Antibodies
☒	☐	Eukaryotic cell lines
☒	☐	Palaeontology and archaeology
☐	☒	Animals and other organisms
☒	☐	Human research participants
☒	☐	Clinical data
☒	☐	Dual use research of concern

Methods

n/a	Involved in the study	
☒	☐	ChIP-seq
☐	☒	Flow cytometry
☒	☐	MRI-based neuroimaging

Antibodies

Antibodies used	The following antibodies and dilutions were used (supplier and catalog number are listed in the filed "validation" below):<br><br>Cox2 (primary rabbit antibody,1:500); Cox5 (primary rabbit antibody,1:500); Tom70 (primary rabbit antibody,1:500); Aco1 (primary rabbit antibody,1:5000); Coq1 (primary rabbit antibody,1:200); Coq5 (primary rabbit antibody,1:333); Coq6 (primary rabbit antibody,1:333); Coq9 (primary rabbit antibody,1:400); Coq7 (primary rabbit antibody,1:1000); Tubulin (primary rabbit antibody,1:10000); FLAG-epitope (primary mouse antibody,1:10000); rabbit IgG (peroxidase-conjugated secondary goat antibody,1:10000); mouse IgG (peroxidase-conjugated secondary rabbit antibody,1:10000)
Validation	Cox2 (primary rabbit antibody) Neupert lab, Munich, Germany (PMID: 30091672)<br>Cox5 (primary rabbit antibody) Ott lab, Stockholm, Sweden (PMID: 22703342)<br>Tom70 (primary rabbit antibody) Rapaport lab, Tübingen, Germany (PMID: 30091672)<br>Aco1 (primary rabbit antibody) Pines lab, Jerusalem, Israel (PMID: 30091672)<br>Coq1 (primary rabbit antibody) Clarke lab, Los Angeles, USA (PMID: 15548532)<br>Coq5 (primary rabbit antibody) Clarke lab, Los Angeles, USA (PMID: 14701817)<br>Coq6 (primary rabbit antibody) Clarke lab, Los Angeles, USA (PMID: 12721307)<br>Coq9 (primary rabbit antibody) Clarke lab, Los Angeles, USA (PMID: 17391640)

Coq7 (primary rabbit antibody) Clarke lab, Los Angeles, USA (PMID: 16624818)
Tubulin (primary rabbit antibody) Abcam, ab184970 (https://www.abcam.com/alpha-tubulin-antibody-epr13799-ab184970.html)
FLAG-epitope (primary mouse antibody) Sigma-Aldrich, F3165 (https://www.sigmaaldrich.com/US/en/product/sigma/f3165)
Rabbit IgG (peroxidase-conjugated secondary goat antibody) Sigma-Aldrich, A0545 (https://www.sigmaaldrich.com/US/en/product/sigma/a0545)
Mouse IgG (peroxidase-conjugated secondary rabbit antibody) Sigma-Aldrich, A9044 (https://www.sigmaaldrich.com/US/en/product/sigma/a9044)

Animals and other organisms

Policy information about studies involving animals; ARRIVE guidelines recommended for reporting animal research

Laboratory animals	Drosophila melanogaster males and females were used. The SPoCk-RNAi (#110379) and Mef2-Gal4 (#27390) line were obtained from Vienna Drosophila Resource Center and Bloomington Stock Center, respectively. The w1118 strain was used as control and for backcrossing of transgenic flies. For determination of CoQ content and Mn levels, third instar larvae and adult flies (collected 1-3 days after eclosion) were used. Mitotracker CMXRos staining for mitochondrial transmembrane potential was performed in dissected third instar larvae. Developmental time was scored from first instar larvae to eclosion of adult flies.
Wild animals	The study did not involve wild animals.
Field-collected samples	The study did not involve samples collected from the field.
Ethics oversight	No ethical approval or guidance was required as only invertebrate animals (Drosophila melanogaster) and yeast were used.

Note that full information on the approval of the study protocol must also be provided in the manuscript.

Flow Cytometry

Plots

Confirm that:

- [x] The axis labels state the marker and fluorochrome used (e.g. CD4-FITC).
- [x] The axis scales are clearly visible. Include numbers along axes only for bottom left plot of group (a 'group' is an analysis of identical markers).
- [ ] All plots are contour plots with outliers or pseudocolor plots.
- [x] A numerical value for number of cells or percentage (with statistics) is provided.

Methodology

Sample preparation	Approximately 1×10^6 yeast cells were harvested from liquid cell culture flasks by centrifugation, resuspended in staining solution containing propidium iodide or MitoTracker Deep Red and incubated for 5 min (propidium iodide) or 10 min (MitoTracker Deep Red). For subsequent flow cytometric analysis, cells were harvested by centrifugation and resuspended in phosphate buffered saline.
Instrument	Guava easyCyte 8HT B/R, Luminex/Merck group
Software	InCyte v3.1
Cell population abundance	For both, propidium iodide and MitoTracker Deep Red staining, 5000 yeast cells per sample (n=1) were analyzed.
Gating strategy	Gating strategies for flow cytometry are shown in Supplementary Fig. 5a, b.<br>For quantification of live and dead cells using propidium iodide (PI), yeast cells were distinguished from debris by gating cells in Side Scatter height (SSC-H) vs Forward Scatter height (FSC-H). Doublets were discriminated from singlets by gating cells in Side Scatter height (SSC-H) vs width (SSC-W). For cell death analysis, this population was gated into PI-positive (dead) and PI-negative (live) populations. Percentage of PI-negative cells is reported as survival (Fig. 1d and 3b) and percentage of PI-positive cells is reported as cell death (Supplementary Fig. 1f). For flow cytometric quantification of mitochondrial mass using MitoTracker Deep Red staining, singlet cells were identified by gating cells in Forward Scatter Area (FSC-A) vs FSC-H. Subsequently, the median red fluorescence intensity of the singlets population was divided by the FSC-H value to normalize to cell size. Gating strategy corresponds to flow cytometry data shown in Supplementary Fig. 1c.

- [x] Tick this box to confirm that a figure exemplifying the gating strategy is provided in the Supplementary Information.
